# Efficient generation of hepatic cells from mesenchymal stromal cells by an innovative bio-microfluidic cell culture device

**DOI:** 10.1186/s13287-016-0371-7

**Published:** 2016-08-19

**Authors:** Meng-Hua Yen, Yuan-Yi Wu, Yi-Shiuan Liu, Marilyn Rimando, Jennifer Hui-Chun Ho, Oscar Kuang-Sheng Lee

**Affiliations:** 1 Institute of Clinical Medicine, National Yang-Ming University, No. 155, Sec. 2, Linong Street, Taipei, 112 Taiwan (Republic of China); 2 Stem Cell Research Center, National Yang-Ming University, No. 155, Sec. 2, Linong Street, Taipei, 112 Taiwan (Republic of China); 3 Taiwan International Graduate Program, National Yang Ming University and Academia Sinica, No. 155, Sec. 2, Linong Street, Taipei, 112 Taiwan (Republic of China); 4 Center for Stem Cell Research, Wan Fang Hospital, Taipei Medical University, No. 111, Section 3, Hsing-Long Rd, Taipei, 116 Taiwan (Republic of China); 5 Graduate Institute of Clinical Medicine, Taipei Medical University, No. 250 Wuxing Street, Taipei City, 110 Taiwan (Republic of China); 6 Department of Ophthalmology, Wan Fang Hospital, Taipei Medical University, No. 111, Sec. 3, Hsing-Long Rd, Taipei, 116 Taiwan (Republic of China); 7 Department of Medical Research, Taipei Veterans General Hospital, No. 201, Sec. 2, Shipai Rd, Beitou District, Taipei City, 112 Taiwan (Republic of China); 8 Taipei City Hospital, No. 145, Zhengzhou Rd, Datong Dist., Taipei, 103 Taiwan (Republic of China)

**Keywords:** Hepatocyte, Mesenchymal stromal cells, Hepatic differentiation, Microfluidic device

## Abstract

**Background:**

Mesenchymal stromal cells (MSCs) are multipotent and have great potential in cell therapy. Previously we reported the differentiation potential of human MSCs into hepatocytes in vitro and that these cells can rescue fulminant hepatic failure. However, the conventional static culture method neither maintains growth factors at an optimal level constantly nor removes cellular waste efficiently. In addition, not only is the duration of differentiating hepatocyte lineage cells from MSCs required to improve, but also the need for a large number of hepatocytes for cell therapy has not to date been addressed fully. The purpose of this study is to design and develop an innovative microfluidic device to overcome these shortcomings.

**Methods:**

We designed and fabricated a microfluidic device and a culture system for hepatic differentiation of MSCs using our protocol reported previously. The microfluidic device contains a large culture chamber with a stable uniform flow to allow homogeneous distribution and expansion as well as efficient induction of hepatic differentiation for MSCs.

**Results:**

The device enables real-time observation under light microscopy and exhibits a better differentiation efficiency for MSCs compared with conventional static culture. MSCs grown in the microfluidic device showed a higher level of hepatocyte marker gene expression under hepatic induction. Functional analysis of hepatic differentiation demonstrated significantly higher urea production in the microfluidic device after 21 days of hepatic differentiation.

**Conclusions:**

The microfluidic device allows the generation of a large number of MSCs and induces hepatic differentiation of MSCs efficiently. The device can be adapted for scale-up production of hepatic cells from MSCs for cellular therapy.

**Electronic supplementary material:**

The online version of this article (doi:10.1186/s13287-016-0371-7) contains supplementary material, which is available to authorized users.

## Background

Liver transplantation is the only definitive treatment for end-stage liver diseases [1]. However, the lack of donor organs has been a major obstacle to the treatment modality. Besides, the long-term use of immuno-suppressants after liver transplantation also brings undesired adverse effects [2]. For these reasons, alternative treatment options are desired. Cell therapy, which offers a potential solution to the problem of donor shortage, has become highly attractive in recent years along with the rapid progress in stem cell technology. However, the unsolved ethical concerns and controversies over the use of human embryos to derive embryonic stem cells (ESCs) have substantially hampered the progress of their clinical applications, not to mention that ESCs are allogeneic in nature and may require further immue-suppression treatment after implantation. Mesenchymal stromal cells (MSCs), which can be obtained from a number of somatic tissues including adipose tissue [3, 4], amniotic fluid [5, 6], placenta [7], and umbilical cord blood [8], are easily available. We have previously reported the differentiation potential of human MSCs into hepatocytes in vitro based on a novel two-step protocol [8, 9]; these cells can rescue fulminant hepatic failure induced by CCl_4_ [10]. Recently, a number of studies have used similar protocols for hepatic differentiation of MSCs based on our previous reports [11–14]. About 4–6 weeks are required to complete the hepatic differentiation process with these protocols. In addition, the need for a large number of hepatocytes for cell therapy has not been addressed fully in these studies.

Shear stress and fluid friction force generated from continuous fluid flow may significantly affect hepatic differentiation of MSCs because liver regeneration in vivo is related to portal pressure, reflecting fluid shear stress [15]. However, very few studies have examined the effects of fluid shear stress on hepatic differentiation. Therefore, it is valuable to investigate hepatic differentiation of MSCs under continuous fluid flow using a microfluidic device which mimics the shear flow in the microenvironment. Microfluidic devices have been utilized in cell culture [16–18], cell differentiation [19–22], dynamic gene expression [23, 24], and test of cellular response to chemical gradients [25–27]. Studies of hepatic differentiation from MSCs using microfluidic devices [20] and bioreactors [28–40], together with 3D scaffolds [41], have been reported. However, there are shortcomings when using conventional microfluidic devices to promote hepatic differentiation. First, an uneven flow in a small culture chamber may affect the quality and yield of differentiated cells [20]. Second, cell injection through seeding microchannels causes an uneven cell distribution which could affect cellular interaction and therefore influence differentiation [17, 19, 20, 26, 42–48]. In addition, when cell injection is completed during the injection processes, some cells still remain in the injection microchannel and are not delivered into the culture chamber. Thus, it is imperative to develop an innovative microfluidic device that minimizes these limitations.

To solve the aforementioned problems, we developed a novel bio-microfluidic device system to enhance the hepatic differentiation capacity of MSCs. The microfluidic device can also improve cell distribution and scale-up cell productions. We further compared the performance of the new system with that of the classic system.

## Methods

### Design and fabrication of the microfluidic device and culture system

**Fig. 1:**
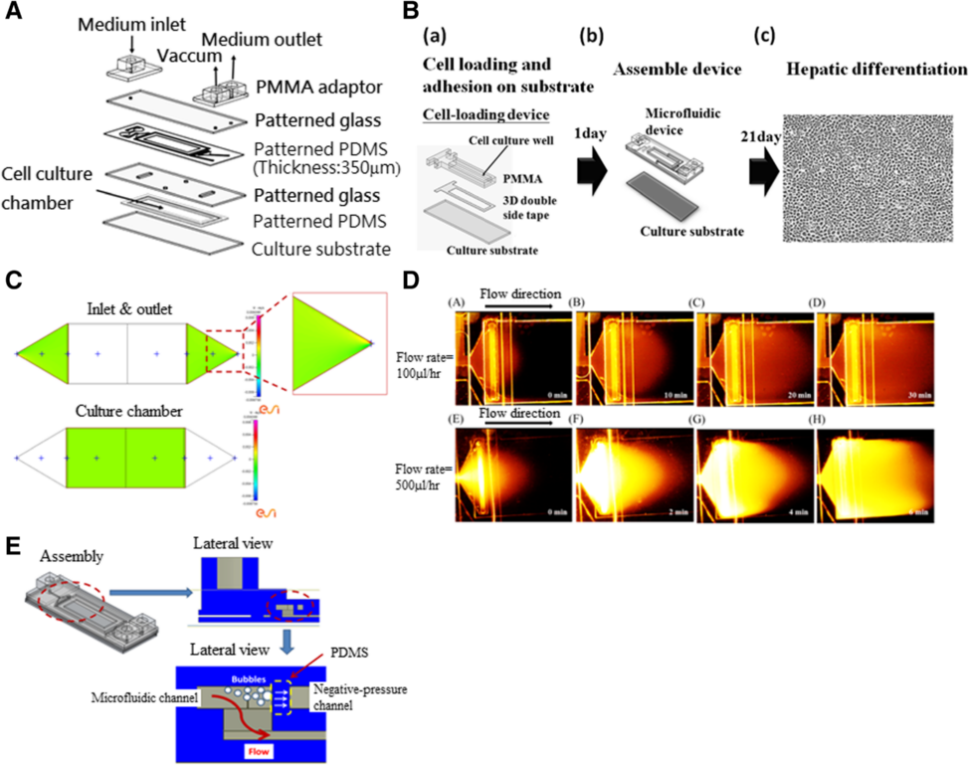
Design of the microfluidic device and flow field rate simulation. **a** Components of the microfluidic device assemblage for cell culture and induction of hepatic differentiation of MSCs. **b** Schematic illustration of the cell culture procedure to induce hepatic differentiation using the microfluidic device. Cells were initially seeded unto the cell-loading device and allowed to adhere onto the substrate (*a*). After 24 hours, the cell loading device was aseptically removed and the substrate was assembled with the other microfluidic components (*b*), cultured in hepatic induction medium and monitored by time-lapse microscopy (*c*). **c** Simulation of medium diffusion in a pre-established flow field. The flow field (*green*) shows a uniform flow profile in the culture chamber of the device. **d** Actual flow field area subjected to two flow rates (100 and 500 μl/hour) during culture medium replacement. The medium flow rate of 100 μl/hour from left to right starts upon injection of the culture medium. Addition replacement at *t* = 0 minutes (*a*), replacement progression at *t* = 10 minutes (*b*) and *t* = 20 minutes (*c*), and complete replacement at *t* = 30 minutes. The flow rate of 500 μl/hour (*lower panel*) shows start of the replacement, *t* = 0 minutes (**e**). Replacement progression at *t* = 2 minutes (*f*) and *t* *=* 4 minutes (*g*). Complete replacement at *t* = 6 minutes (*h*). **e** Schematic representation in lateral view of air bubble removal by negative pressure during injection of culture medium into the cell culture chamber. *PDMS* polydimethylsiloxane, *PMMA* polymethyl methacrylate

The features of the microfluidic device include an open-cover design, use of a laser direct writing (LDW) technique, an air bubble-removal design, and a choice of materials. An open-cover design, in which the microfluidic device and the culture substrate with a patterned cell culture region were sealed by negative pressure, was adopted in order to homogeneously distribute the cells onto the culture substrate. A cell loading device fabricated by a LDW technique (CO_2_ laser machine, ILS-II; Laser Tools and Techniques, Hsinchu, Taiwan) was performed to pattern the cell culture region to ensure a good match between the cell culture region and the culture chamber (Fig. 1b). The cells were then seeded by the conventional method and allowed to attach onto the substrate. After attaching the cells to the substrate, the loading device was removed and the microfluidic device was assembled. The assembly procedure was simple and the setup time was less than 5 minutes. To assure a long-term culture without interference, an air bubble-removal structure in the microchannel was used to avoid injection of air bubbles into the culture chamber. Besides, the transparent feature of our device allowed the cell morphology to be observed easily in real time. In brief, this device enables a homogeneous cell distribution, large culture chamber, rapid development time, rapid operation time, and real-time observation under an optical microscope.

The microfluidic device was designed to have a culture chamber dimension of 10 mm × 40 mm × 350 μm (width × length × height), with a culture area of 400 mm^2^. The device was assembled in five layers (Fig. 1) consisting of a lower layer of a culture substrate, on top of an intermediate layer formed by two patterned glass and two patterned polydimethylsiloxane (PDMS) membranes (Sylgard 184; DowCorning, Midland, MI, USA), with a top layer of polymethyl methacrylate (PMMA), including three adaptors for producing the vacuum, medium inlet, and outlet. The PDMS membranes were prepared and fabricated according to the manufacturer’s instructions. These PDMS membranes were patterned by a CO_2_ laser machine and the glass was patterned by an ultrasonic drilling machine (LUD-1200; Lapidary & Sonic Enterprises, Taipei, Taiwan). The substrate was made from a polystyrene plate (PS) (25 mm × 75 mm) cut from a culture dish using a CO_2_ laser. Finally, the patterned glass and PDMS were bonded together by a plasma treatment system (PX-250; Nordson, Westlake, OH, USA) and stuck to the PMMA adaptor with double-sided tape to completely assemble the microfluidic device. The microfluidic device, which included a cell culture chamber, a vacuum, and air bubble trap regions, was placed on top of the PS culture substrate. The function of the vacuum region was to seal the culture substrates within the microfluidic device by negative pressure. The pressure applied for sealing is about 85 mmHg. For future large-scale studies, the culture chamber can be further scaled up (up to now, its maximal culture area is 32,400 mm^2^, as shown in Additional file 1: Figure S1). In addition, the device was sterilized by γ-ray radiation before the experiments.

**Fig. 2:**
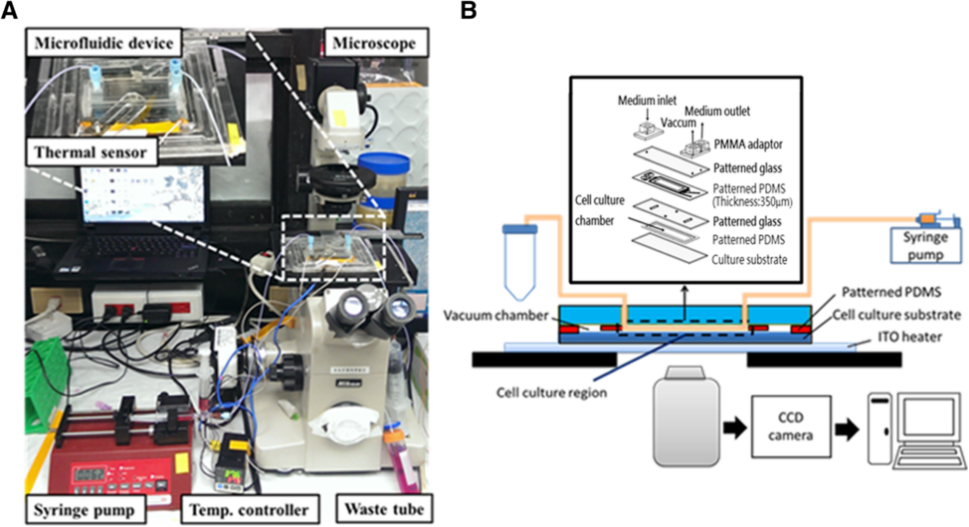
Assemblage of the complete microfluidic system for cell culture and time-lapse observation of MSC hepatic differentiation. **a** Actual microfluidic system for cell culture. *Insert* shows the presence of a thermal sensor attached to the microfluidic device for temperature regulation. **b** Developed microfluidic system. The culture system including the designed microfluidic device consists of a temporal sensor, a syringe pump, a temperature controller, one inlet connecting the syringe unto the device, one outlet connecting waste tube, and a vacuum. *PDMS* polydimethylsiloxane

The assembled microfluidic culture system included the actual microfluidic device with a thermal sensor and regulator, a syringe pump, an inlet connecting the syringe for culture medium injection, a separate outlet connected to the waste tube, and a vacuum (Fig. 2a, b). The device was connected to a time-lapse microscope for real-time observation, attributed to the transparency of the device chamber. The temperature controller ensures a stable temperature of the culture chamber. The syringe pump supplied fresh medium into the system, and the time-lapse microscope allowed real-time observation of the cellular morphology of MSCs during hepatic differentiation.

### Cultivation of MSCs

MSCs were harvested from the bone marrow of postnatal 7-week-old C57BL/6 J mice (National Laboratory Animal Center, Taipei, Taiwan). Approval for the experiment was obtained from the Taipei Veterans General Hospital Institutional Animal Care and Use Committee (IACUC) regarding the use of animals prior to commencement of the experiments. For maintenance and culture expansion, MSCs were maintained in Dulbecco’s modified Eagle’s medium with 1000 mg/L glucose (LG-DMEM; Sigma-Aldrich, St. Louis, MO, USA) supplemented with 10 % fetal bovine serum (FBS; Gibco Invitrogen, Carlsbad, CA, USA), 100 units/ml penicillin, 100 μg/ml streptomycin, 2 mM l-glutamine (Gibco Invitrogen), 10 ng/ml basic fibroblast growth factor (bFGF; Sigma-Aldrich), and 10 ng/ml epidermal growth factor (EGF; R&D Systems, Minneapolis, MN, USA). Cells were seeded at a density of 3 × 10^3^ cells/cm^2^ (30–40 % confluence). They were subcultured and expanded when reaching 80–90 % confluence. Confluent cells were detached with 0.1 % trypsin-EDTA (Gibco Invitrogen), rinsed twice with PBS, and centrifuged at 200 × *g* for 5 minutes. Cell pellets were rinsed twice with PBS and resuspended in culture medium. The cells were re-seeded at a density of 8 × 10^3^ cells/cm^2^ prior to hepatic differentiation under the same culture conditions. The culture medium was replaced three times a week. All cultures were maintained at 37 °C in a humidified atmosphere containing 5 % CO_2_.

### Proliferation and hepatic differentiation of MSCs on the microfluidic device

The procedures for proliferation and hepatic differentiation of MSCs on the culture dish and the microfluidic device are described in the supplementary material (Additional file 1: Figure S2). Hepatic differentiation was initiated using the two-step protocol we reported previously [9]. Mouse MSCs were used for hepatic differentiation and therefore the differentiation time is about 3–4 weeks [49]. Step-1 induction medium, consisting of Iscove’s modified Dulbecco’s medium (IMDM; Gibco BRL, Grand Island, NY, USA) supplemented with 20 ng/ml hepatocyte growth factor (HGF; R&D Systems), 10 ng/ml bFGF, 0.61 g/L nicotinamide (Sigma-Aldrich), and 100 units/ml penicillin, 100 μg/ml streptomycin, 2 mM l-glutamine, was used for induction in the first 7 days. Step-2 maturation medium, consisting of IMDM supplemented with 20 ng/ml oncostatin M (ProSpec, East Brunswick, NJ, USA), 1 μmol/L dexamethasone (Sigma-Aldrich), and 50 mg/ml insulin–transferrin–selenium (6.25 mg/ml insulin, 6.25 mg/ml transferrin, 6.25 ng/ml selenious acid, ITS^+^ premix; Becton Dickinson, Franklin Lakes, NJ, USA), was used for induction for 2 weeks. During the hepatic differentiation, induction medium was supplied from the syringe and injected into the chamber of the microfluidic device through the pipeline, and the outlet was connected to the waste tube. Cellular waste products were removed continuously inside the chamber. The flow rate was 100 μl/hour. For the control group, MSCs were cultured on the PS without continuous flow and were induced by the same protocol.

### Functional analysis, flow field simulation, and statistical analysis

Details of the materials and methods used for RNA extraction, quantitative real-time PCR, immunofluorescent staining, flow cytometry analysis, uptake of low-density lipoprotein (LDL), urea production assay, flow field simulation and observation, and statistical analysis are described in Additional file 1.

## Results

### Design, assembly, and performance test of the bio-microfluidic culture system

A five-layered microfluidic device assembled from one layer of PS, patterned PDMS, patterned glass, and PMMA adaptors was designed and fabricated (Fig. 1a). Together with the other components of the bio-microfluidic system, the device was effectively utilized for the maintenance and hepatic differentiation of MSCs (Figs. 1b and 2). Since the force generated by the flow rate of the culture medium can influence the proliferation and differentiation of MSCs, the perfusion performance in our system was initially simulated and tested (Fig. 1c, d). We aimed at maintaining a constant flow rate and homogeneous medium flow of media in the cell culture chamber. The simulated field of velocity distribution is illustrated in Fig. 1c. The simulation result showed a uniform flow profile in the culture region of the microfluidic device. In addition, the distribution of rhodamine showed that the flow field near the center line and the margin was uniform (Fig. 1d) and matched the simulation result. The Reynolds number (Re) of our microfluidic device for a 100 μl/hour flow rate was 3.6 × 10^–5^. The flow is considered laminar flow when Re is smaller than 2100, and turbulent flow when larger than 4000 [50]. The 100 μl/hour flow rate in our device was therefore a laminar flow.

Efficiency of air bubble removal was further validated. The air bubble-removal structure based on the principle of negative pressure prevented experimental failures and inconsistencies of experimental data caused by air bubble interferences inside the culture chamber. Figure 1e is the schematic representation of the air bubble-removal structure. The material of the barrier wall between the microfluidic channel and the negative-pressure channel was PDMS. When air bubbles enter the microfluidic channel along with culture medium, they can be removed by the air bubble-removal structures by applying negative pressure through the gas-permeable PDMS. A movie of this process demonstrates the slow disappearance of a large air bubble injected into the microchannel of the device 12 hours previously (see Additional file 2: Video 1). The result showed that the “bubble traps” within the device were effective for removing the air bubbles. After validation of the quality of the device and performance of the perfusion system, the microfluidic device was used for the subsequent experiments of proliferation and differentiation of MSCs.

### Morphology, proliferation, pH, and immunophenotype of MSCs cultured under the microfluidic device

**Fig. 3:**
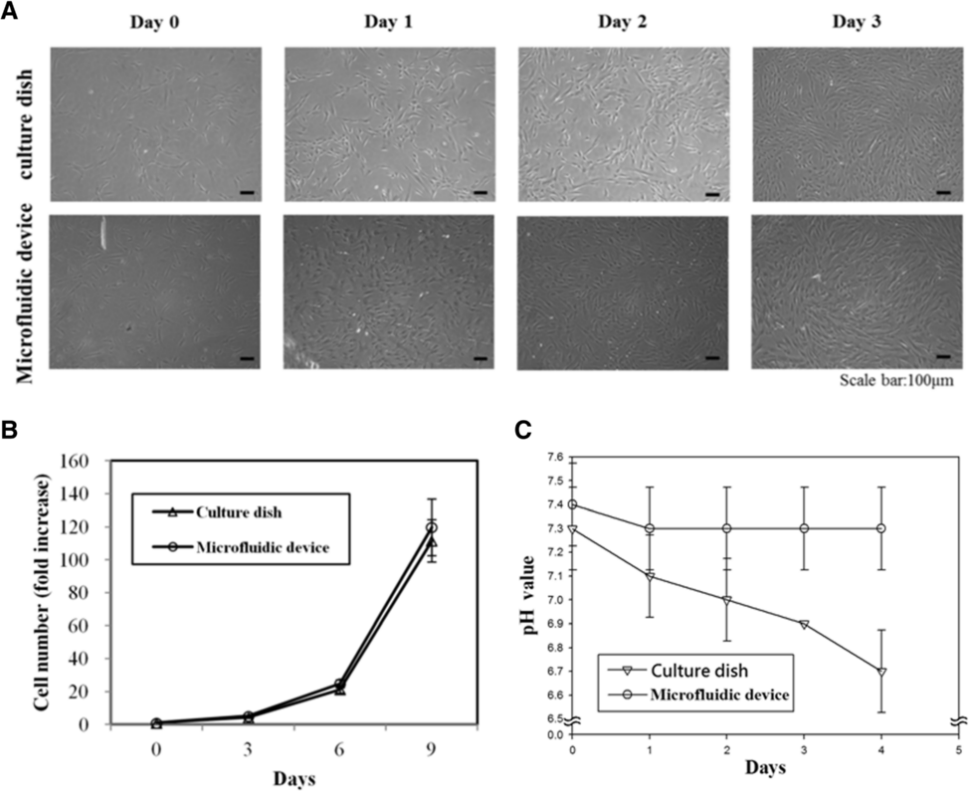
Cell morphology and growth kinetics of MSCs cultured in the culture dish and microfluidic device. **a** MSCs were seeded at a density of 3 × 10^3^ cells/cm^2^ in the microfluidic device and culture dish for 3 days. The cell morphology was compared between the two groups and showed that the cells exhibited a well-adhered and healthy morphology in both groups. *Scale bar* = 100 μm. **b** Growth population curve of MSCs cultured in the culture dish and microfluidic device showed that the growth curves of MSCs cultured in both groups were almost similar from 0 to 9 days. **c** The pH value of the culture medium was monitored, measured, and compared within 4 days in both groups. The pH value in the static culture dish group gradually decreased over time, while the pH value in the microfluidic system remained constant after several days of culture

Prior to the induction of hepatic differentiation of MSCs, the proliferation and expression of stemness markers of MSCs of cells cultured in the microfluidic device and the culture dish were validated. After 3 days of culturing, the cells showed a well-adhered and healthy morphology in both the microfluidic device and the culture dish (Fig. 3a). Also, the cell distribution on PS was homogeneous in both groups. The growth curves of mouse MSCs were similar in both the culture dish and the microfluidic device after 9 days of culture (Fig. 3b). However, we did observe a significant difference in proliferation rates of human MSCs between those cultured in the culture dish and those cultured in the microfluidic device under different flow rates (Additional file 1: Figure S3). We concluded that the cell viability of mouse MSCs cultured in the microfluidic device was no different from that of MSCs cultured in the culture dish.

Previous studies have shown that an optimum pH value is critical for cells to achieve high confluence and efficient enzyme activity [43]. The pH level of the culture medium in the microfluidic device and culture dish was therefore measured during the first 3 days of culturing. The result showed that the pH value of the medium in the microfluidic device was maintained at about 7.3, but decreased from 7.4 to 6.8 in the culture dish and continued to decrease over time (Fig. 3c).

**Fig. 4:**
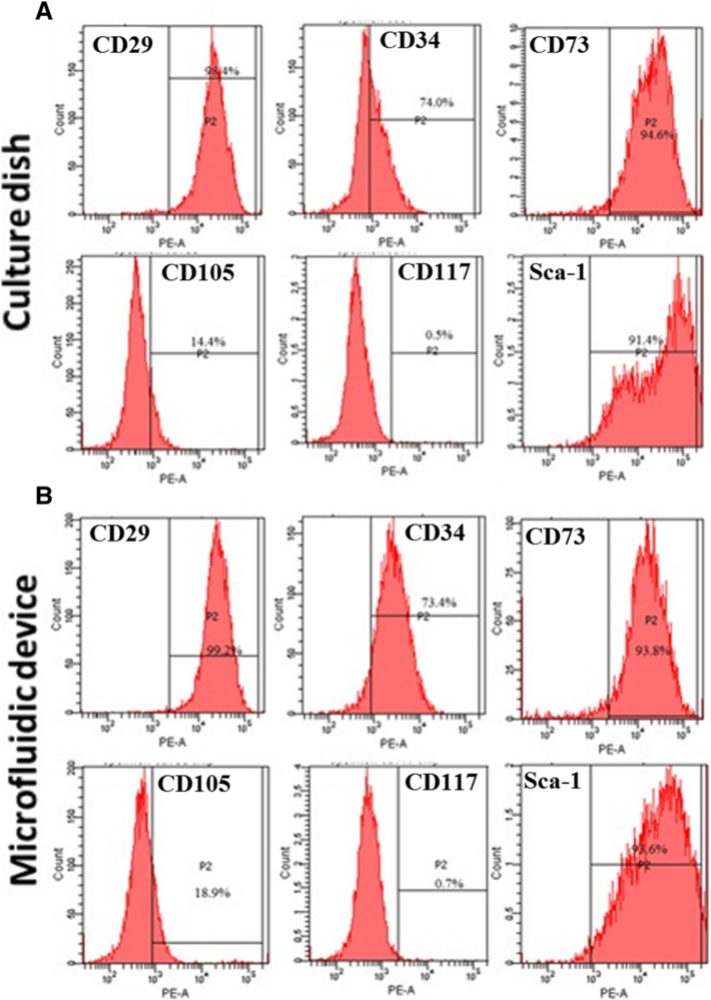
Immunophenotype analysis of mouse MSCs cultured in the culture dish and microfluidic device. Expression of surface markers in mouse MSCs cultured in the **a** static culture dish and **b** microfluidic device at day 3 were validated using flow cytometry. Representative histograms confirmed that the MSCs did not express antigen CD34 but expressed stem cell surface markers CD29, CD105, and SCA-1

To investigate whether the microfluidic system has an influence on MSC phenotype during maintenance, the expressions of cell surface markers of MSCs after 3 days of culture in the microfluidic device and culture dish were analyzed by flow cytometry. Figure 4 shows that most MSCs cultivated in microfluidic devices and culture dishes expressed the standard MSC surface markers, such as CD29 and Sca-1. Although mouse MSC surface markers are previously known to be CD34-negative and CD105-positive cells, studies have shown that certain mouse MSCs are CD34-positive [51]; in addition, CD105-positive and CD105-negative mouse MSCs represent two independent subpopulations that maintain their properties upon several passages [52]. The mouse MSCs we used in this study are therefore another bone marrow subpopulation (CD34-positive and CD105-negative). Moreover, the results showed that the surface markers of MSCs remained unchanged after the 3-day culture in the microfluidic system. In addition, we tested the expression of surface markers in MSCs cultured in the static culture dish and microfluidic device at day 0. Comparative expression of surface markers in mouse MSCs cultured in the static culture dish and microfluidic device at day 0 and day 3 are shown in Additional file 1: Figure S4. The results demonstrate that the expression of surface makers at day 0 is almost the same as that at day 3. MSCs cultured in both environments were therefore similar and both have the potential to further differentiate into hepatocytes. Because the expressions of MSC surface markers in the microfluidic system and the static culture system were similar, a comparison of hepatic differentiation between the two systems was dependable.

### Hepatic differentiation of MSCs in the microfluidic device

**Fig. 5:**
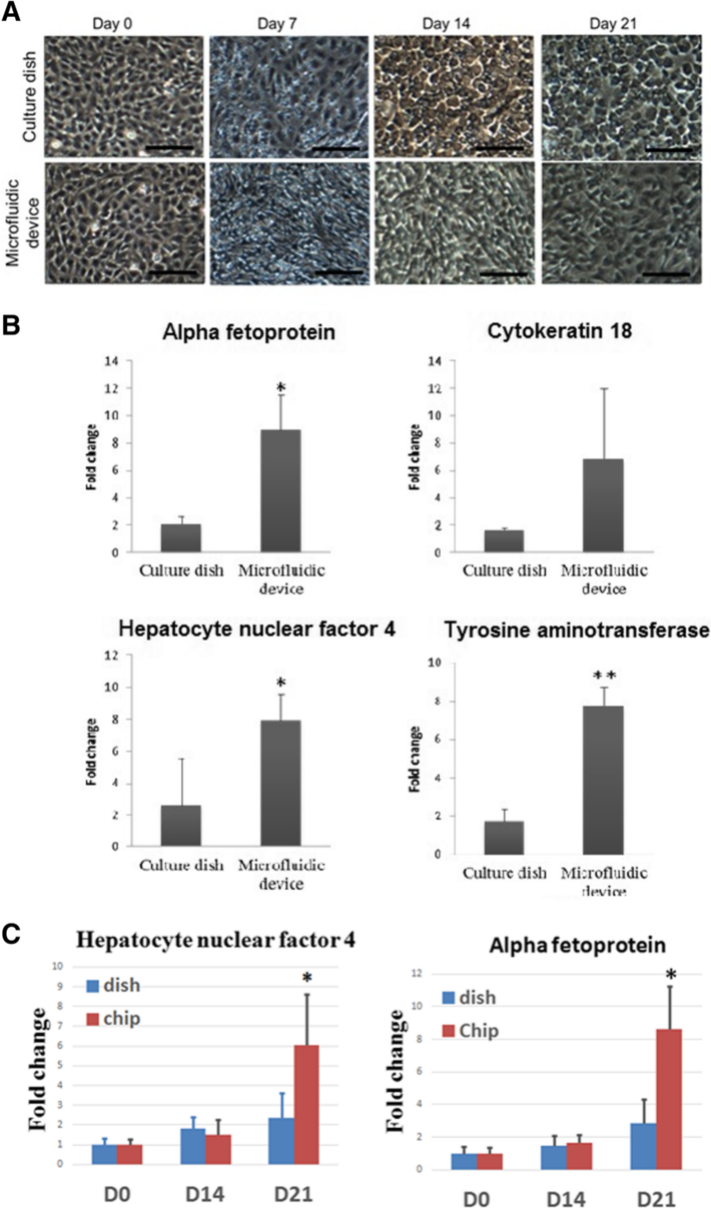
Morphology and gene expression profile of hepatic differentiated MSCs using the microfluidic system. **a** MSCs were grown in the culture dish and microfluidic device and were allowed to differentiate into hepatocyte-like cells within 21 days using the two-step hepatic induction protocol. Cells were observed under a light microscope. *Scale bar* = 100 μm. **b** Comparative gene expression of hepatic differentiated MSCs in the culture dish and the microfluidic device after 21 days of two-step induction. Hepatocyte-like cells derived from MSCs in the microfluidic system expressed higher hepatocyte-specific marker genes than those in the culture dish. Data presented as mean ± SD (*n* = 3). *P < 0.05, **P < 0.01, Student’s *t* test. **c** Comparative gene expression of hepatic differentiated MSCs in the culture dish and the microfluidic device during 21 days. Gene expressions of *HNF4* and *AFP* at day 14 between the dish group and the microfluidic device group are similar. Data presented as mean ± SD (*n* = 3). *P < 0.05, **P < 0.01, Student’s *t* test

The cellular morphology, gene expression level, protein level, and functional activity of differentiated hepatocyte-like cells during hepatic MSC differentiation in the microfluidic device were compared with those in the culture dish. Cellular morphology was observed during the first 7 days of hepatic differentiation using step-1 induction medium. The morphology of MSCs in both the microfluidic device and the culture dish showed an elongated and spindle-like shape form during the first 7 days of induction. The cellular morphology of MSCs gained a cuboidal shape after 14 days of step-2 medium induction both in the device and in the dish (Fig. 5a). This result indicated that the cellular morphology of MSCs was not influenced by flow generated in the microfluidic device culture system. Furthermore, the time-lapse movie (see Additional file 3: Video 2) shows no difference of cellular morphology during 21 days of hepatic differentiation between cells cultured in the device and in the culture dish.

**Fig. 6:**
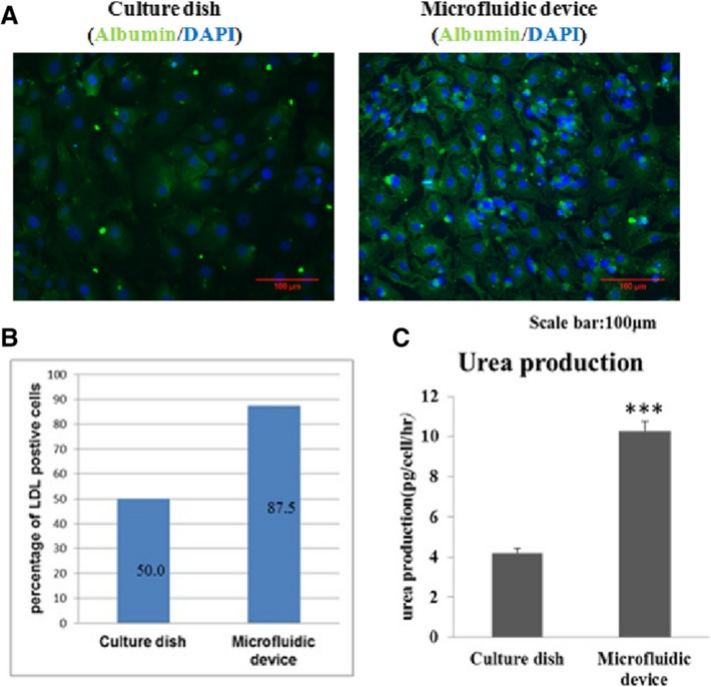
Protein expression of differentiated hepatocyte like-cells from MSCs in the culture dish and microfluidic device. Expression of a marker of functional hepatocyte (albumin) was assessed by immunofluorescence staining from differentiated hepatocyte-like cells after 21 days of induction (**a**). Cells were observed under a light microscope at 200× magnification. *Scale bar* = 100 μm. In addition, comparison of LDL uptake and urea production of MSCs cultured in the (**b**) culture dish and (**c**) microfluidic device are shown. MSCs were fixed at day 21 and stained with LDL uptake in both cell groups. The percentage of LDL-positive MSCs at day 21 is shown in (**b**). LDL uptake was higher in the MSCs cultured in microfluidic device compared with those cultured in the culture dish. (**c**) After 21 days of two-step induction, higher urea production was observed in hepatocyte-like cells growing in the microfluidic device than in the culture dish. Data presented as mean ± SD (*n* = 3). ***P < 0.001, Student's *t* test. *DAPI* 4′,6-diamidino-2-phenylindole. *LDL* low-density lipoprotein

To investigate whether there were significant differences in gene expression of hepatic markers between the two environments, the expressions of hepatocyte-associated genes were detected by RT-PCR at day 21 of hepatic differentiation of MSCs. The result showed that the expressions of liver-associated genes were upregulated in both environments (Fig. 5b). The hepatic differentiation of MSCs in the microfluidic device showed an almost 4-fold to 5-fold increase of liver-associated gene expression compared with that in the culture dish (Fig. 5b). In addition, Fig. 5c shows the comparative gene expression of hepatic differentiated MSCs in the culture dish and the microfluidic device during 21 days. The result shows that the gene expressions of hepatocyte nuclear factor 4 (*HNF4*) and alpha fetoprotein (*AFP*) at day 14 between the dish group and the microfluidic device group are similar. This means that the hepatic differentiation in the microfluidic device is not delayed. Also, differences of protein expression between the two environments were investigated. Immunofluorescence staining for albumin after 21 days of hepatic differentiation in the two cultural systems is shown in Fig. 6a. The fluorescence intensity of albumin in the microfluidic device was stronger compared with that in the culture dish. Furthermore, LDL uptakes from differentiated MSCs in both environments were assessed by staining the cells at day 21 with LDL assay dye. The number of LDL-positive cells were counted and compared. As shown in Fig. 6b, the percentage of LDL-positive MSCs from the microfluidic device was higher than that from the culture dish. Based on previous studies showing that functional hepatocytes are capable of producing urea [53], a urea functional assay was used to assess the bio-activities of hepatocyte-like cells (Fig. 6c). The result showed that MSCs in the microfluidic device produced 2.5-fold more urea than that in the culture dish. Collectively, based on these results, we concluded that the bio-microfluidic system efficiently generated more differentiated hepatocyte-like cells from MSCs compared with the conventional static culture.

## Discussion

In this study, a perfusion-based microfluidic system was designed to enhance the production of differentiated hepatocytes from MSCs. The design showed substantial advantage in promoting hepatogenesis of MSCs. Our design has two major advantages over other similar devices which use negative pressure to seal the open cover, such as the hybrid microfluidic–vacuum system [54, 55]. First, the use of LDW techniques reduced the fabrication time of our microfluidic system from days to hours compared with the conventional micro-electromechanical techniques [48]. Also, the fabrication of a large culture chamber is made easy with the help of LDW techniques. Second, unlike other previous designs [54, 55], cells are not pressed against the microfluidic device at the edges of culture chamber during assembly due to a close match between the cell culture region and the culture chamber in our system; this design prevents cell damage during assembly. In addition, the microfluidic device can be reversibly bonded and unbonded to the culture substrate, which is useful for further immunocytochemistry or manipulation of cell colonies. For example, unwanted colonies can be removed easily without affecting the healthy ones during maintenance. This device may also be suitable for the differentiation of ESCs or induced pluripotent stem cells (iPSCs).

Besides a larger cell culture surface area, a geometry that allows uniform flow distribution within the culture chamber is also a featured characteristic which confers several advantages for hepatic differentiation of MSCs. The culture chamber of our microfluidic device (culture area ≥ 400 mm^2^) was significantly larger than that previously reported (culture area = 7 mm^2^) [20]. Incorporating this device into an automated system, scale-up production for cell therapy is possible. Moreover, the flow field distribution of the culture medium inside the chamber is more uniform because of the quadrilateral geometry of the culture chamber, because the flow field of a rectangular chamber creates a more uniform flow (Fig. 1c, d). On the contrary, uneven flow occurs in a circular chamber (Additional file 1: Figure S5). Hence, a more homogeneous flow distribution within the culture chamber can be achieved in our device, which offers a favorable environment for hepatic differentiation of MSCs.

The microfluidic system, which mimics the shear flow microenvironment, theoretically confers advantages over the static culture system. It supplies a continuous flow of fresh medium and facilitates constant removal of metabolic wastes, which provides a constant microenvironment to maintain cell viability and function over a longer culture period. On the contrary, there are inherent limitations in the traditional in-vitro static culture systems, such as nutrient supply and metabolic end-product accumulation, including release of toxins that negatively influences the cell growth [56]. The utilization of our bio-micofluidic device with a favorable environment efficiently facilitates and accelerates hepatic differentiation of MSCs. In addition, a previous study has demonstrated that the microenvironment is essential for the stemness properties of stem cells [57]. The cellular microenvironments of MSCs in the microfluidic device and the culture dish were similar, and so was the cell viability of MSCs.

The microenvironment in the culture chamber is the key for efficient hepatic differentiation of MSCs in this study. The continuous flow not only constantly supplies nutrients for hepatic differentiation but also removes the unhealthy cells that loosely attached onto the culture chamber. As a consequence, hepatocyte-like cells with homogeneous and favorable functional activities are obtained. A previous study indicates that removal of cell-secreted factors suppresses cell growth and differentiation [58]. However, this action did not seem to suppress cell growth and differentiation in our study. This might be due to the use of a two-step hepatic differentiation protocol which offers necessary growth factors to support hepatogenesis [9]. These factors can be maintained at a relatively stable and higher concentration in the microfluidic system compared with the static culture. A higher expression of hepatic marker genes in the microfluidic device group may be attributable to the mechanical microenvironment as well as more homogeneous cell distribution in the device.

Shear stress plays a crucial role in regulating proliferation, differentiation, and cellular morphology of MSCs [59–61]. The shear stress generated in our microfluidic device was much lower (0.00142 Dyne/cm^2^) than those previously reported to regulate the proliferation and osteogenic differentiation of MSCs (0.3–2.7 Dyne/cm^2^) [59–61]. It did not affect the lineage commitment of MSCs because there were no significant difference between the microfluidic system and static culture in surface immunophenotype after 3 days of maintenance (Fig. 4). This means that shear stress was not a key factor for stemness properties of MSCs in our study. Moreover, a previous study indicates that low shear stress could further stimulate maturation signals through sensory systems on polarized hepatic cells [62]. Indeed, higher expression of hepatic marker genes was observed in the microfluidic device and the mechanical signals might have contributed to functional maturation of hepatocyte-like cells.

Based on our results, MSCs in the microfluidic device after 3 weeks of hepatic induction expressed a high level of *AFP* and *HNF4*. Because *AFP* is specifically expressed in hepatocyte progenitors, it is therefore one of the indicators for hepatic differentiation [63, 64]. The high level of *AFP* after 3 weeks of hepatic differentiation means that use of the microfluidic device can accelerate the hepatic differentiation. The results indicated that both hepatocyte progenitors (*AFP* high expression) and matured hepatocytes (*HNF4* high expression) in the microfluidic device are greater than those in the culture dish, due to the optimal differentiation conditions. Since previous studies have indicated that hepatic progenitor cells (or fetal liver cells) possess greater regeneration potential than fully matured hepatocytes in experimental liver failure models [49, 65, 66], the cells differentiated in the microfluidic device should be suitable for cell therapy compared with those generated from 2D static culture. The hepatic differentiation from MSCs in the microfluidic device is therefore a more efficient method than that in the culture dish.

## Conclusions

An innovative microfluidic system that allows homogeneous cell seeding and real-time observation of cell morphology has been developed. The system efficiently generates hepatocyte-like cells from MSCs with more rapid functional maturation than a conventional static culture system. This microfluidic system may be further developed for large-scale production of hepatocyte-like cells to meet the high demand in the cell therapy industry. More efforts will be devoted to further scale-up and automation of this novel device.

## Abbreviations

bFGF, basic fibroblast growth factor; EGF, epidermal growth factor; ESC, embryonic stem cell; FBS, fetal bovine serum; HGF, hepatocyte growth factor; IMDM, Iscove’s modified Dulbecco’s medium; iPSC, induced pluripotent stem cell; LDL low-density lipoprotein. Re, Reynolds number; LDW, laser direct writing; LG-DMEM, Dulbecco’s modified Eagle’s medium with 1000 mg/L glucose; MSC, mesenchymal stromal cell; PDMS, polydimethylsiloxane; PMMA, polymethyl methacrylate; PS, polystyrene plate

## Additional files

Additional file 1: Is supplementary materials and methods: Table S1 presenting the sequence of quantitative PCR primers for MSCs, Figure S1 showing the microfluidic device with larger culture chamber used for the study, Figure S2 showing the processes of proliferation (A) and hepatic differentiation (B) of MSCs in the culture dish and microfluidic device, Figure S3 showing the growth curve of human MSCs cultured in the microfluidic device and culture dish from 0 to 9 days, Figure S4 showing the comparative expression of surface markers in mouse MSCs cultured in static culture dish and microfluidic device at day 0 and day 3, and Figure S5 showing the simulation of culture medium diffusion in a circle cultural chamber. The flow field showed an uneven flow profile in a circle cultural chamber. The dimension and parameters of flow field were based on a previous study [20]. (DOCX 979 kb)

Additional file 2: Is Video 1 showing the movie of air bubble removal from the cell culture chamber of the microfluidic device. *Scale bar* = 1 mm. (MP4 1789 kb)

Additional file 3: Is Video 2 showing the time-lapse movie shows the hepatic differentiation from MSCs in the device. *Scale bar* = 100 μm. (MP4 3883 kb)
