# Supplementary material for: Efficient generation of hepatic cells from mesenchymal stromal cells by an innovative bio-microfluidic cell culture device
Source: Stem Cell Res Ther. 2016 Aug 19;7:120. doi: 10.1186/s13287-016-0371-7 (PMC4992324; doi:10.1186/s13287-016-0371-7)
Supplement: Supplementary file 1 — Is supplementary materials and methods: Table S1 presenting the sequence of quantitative PCR primers for MSCs, Figure S1 showing the microfluidic device with larger culture chamber used for the study, Figure S2 showing the processes of proliferation (A) and hepatic differentiation (B) of MSCs in the culture dish and microfluidic device, Figure S3 showing the growth curve of human MSCs cultured in the microfluidic device and culture dish from 0 to 9 days, Figure S4 showing the comparative expression of surface markers in mouse MSCs cultured in static culture dish and microfluidic device at day 0 and day 3, and Figure S5 showing the simulation of culture medium diffusion in a circle cultural chamber. The flow field showed an uneven flow profile in a circle cultural chamber. The dimension and parameters of flow field were based on a previous study [20]. (DOCX 979 kb) [file 13287_2016_371_MOESM1_ESM.docx]

**Additional file 1**

1. **Supplementary materials and methods**

1.1. *Flow field simulation and observation*

For flow field simulation, a computational fluid dynamics software package, CFD-ACE+ (CFD Research, Huntsville, AL, USA) was used to model the flow field inside the cell culture chamber. Rhodamine 6G (No. 419011000, Acros, Fair Lawn, NJ, USA) was used as a tracing dye for the actual flow field observation inside the chamber.

1.2. *Processes of proliferation and hepatic differentiation of MSCs on culture dish and microfluidic device*

Experimental processes of the proliferation and hepatic differentiation from MSC on culture dish and microfludic device are shown in Figure S2. In order to ensure MSCs attach on the substrate well, the first step of the proliferation or hepatic differentiation was to culture MSCs on the substrate one day before the experiments. The seeding density of MSCs for the proliferation or differentiation experiments was 3000 cells/cm^2^ and 8000 cells/cm^2^ respectively. Experimental processes of the proliferation are shown in Figure S2A. MSCs were cultured for three days in a microfluidic device and a 6-cm culture dish. MSC cultured on the substrate and reached 80%-90% confluence at day 3. Then the MSCs were collected for further bioassay. Experimental processes of the hepatic differentiation are shown in Figure S2B. When MSCs were grown to 90% confluence on the PS, the PS was assembled into the microfluidic system. After 21 days of induction, the MSCs were collected for further bioassay. For cell number counting, microfluidic device and substrate were separated by releasing sealing pressure first; cells were then detached using 0.1% trypsin-EDTA and counted cell number by cell counter

1.3. *RNA extraction and quantitative real time polymerase chain reaction*

To detect hepatic gene expression, RNA was isolated using Trizol reagent (Invitrogen, Carlsbad, CA, USA) and 2 µg of RNA samples were reversed transcribed using MMLV Reverse Transcriptase 1st-Strand cDNA synthesis Kit (EPICENTRE^®^ Biotechnologies, Madison, WI, USA). 10 ng of cDNA was loaded per reaction and mixed with Fast SYBR® Green Master Mix 2X (Applied Biosystems, Foster City, CA, USA). qPCR reaction was performed by Step One plus real-time PCR system (Applied Biosystems, Foster City, CA, USA). Glyceraldehyde-3-phosphate dehydrogenase (GAPDH) was used as an endogenous internal control. The primers used for qPCR are listed in Table S1. Several marker genes of hepatocyte, such as alpha-fetoprotein (AFP), glucose 6-phosphatase (G6P), tyrosine aminotransferase (TAT), cytokeratin-18 (CK18) and hepatocyte nuclear factor-4 (HNF4), were examined.

1.4. *Immunofluorescent staining*

For staining of intracellular proteins, the samples were washed with PBS and fixed with 3.7% paraformaldehyde (Sigma-Aldrich, St. Louis, MO, USA) for 20 minutes. After fixation, the cells were washed with PBS and permeabilized with 0.1% Triton X-100 (Sigma-Aldrich, St. Louis, MO, USA) for 20 minutes. After three times of successive washing with PBS, the samples were blocked with 1% bovine serum albumin (BSA, Thermo EC, Milford, MA, USA) for 1 hour and incubated with specific primary antibodies overnight at 4 ºC. The primary antibody was primary monoclonal albumin (1:200)(ALB, for human/mouse use, R&D Systems, Minneapolis, MN, USA) diluted in 0.1% BSA. In the following day, the samples were washed three times with PBS and incubated with secondary antibody (1:200) at room temperature for one hour. After washing thrice with PBS, the samples were incubated with 1:1000 diluted 4',6-diamidino-2-phenylindole (DAPI, Sigma-Aldrich, St. Louis, MO, USA) for 10 minutes, to label the nucleus. Finally, after washing PBS thrice, the samples were covered reversely on a cover slide with 50μl mounting media, and sealed with nail polish. The samples were imaged using an optic microscope (AX80, Olympus, Melville, NY, USA).

1.5. *Flow cytometry analysis*

For flow cytometry analysis, cells cultured in microfluidic device and 6-cm culture dish were harvested with 0.25% trypsin-EDTA, re-suspended with PBS containing 1% serum and aliquoted into fresh 1 ml flow tubes. The cell pellet was centrifuged for 5 minutes at 200×g, and rinsed with PBS containing 1% serum and incubated with PE-conjugated antibodies for 30 minutes in the dark on ice. Next, cells were washed with PBS containing 1% serum and centrifuged for 5 minutes at 200×g. The pellet was re-suspended in 250μl PBS containing 1 % serum and incubated with the following PE-conjugated antibodies: anti-mouse CD29 (1:200, eBioscience, San Diego, CA, USA), anti-mouse CD34 (1:200, BD Biosciences, San Jose, CA, USA), anti-mouse CD44 (1:200, BD Biosciences, San Jose, CA, USA), anti-mouse CD73 (1:200, eBioscience, San Diego, CA, USA), anti-mouse CD105 (1:200, eBioscience, San Diego, CA, USA), anti-mouse CD117 (1:200, BD Biosciences, San Jose, CA, USA), anti-mouse SCA-1 (1:200, BD Biosciences, San Jose, CA, USA). The rat IgG2a kappa (1:200, BD Biosciences, San Jose, CA, USA), rat IgG2b kappa (1:200, BD Biosciences, San Jose, CA, USA), and Armenian Hamster IgG, kappa (1:200, BD Biosciences, San Jose, CA, USA) were used as negative control. Cells were analyzed using a BD FACSC anto™ II Flow Cytometry System and analyzed by BD FACS Diva software (BD Biosciences, San Jose, CA, USA).

1.6. *Uptake of low-density lipoprotein and urea production assay*

The conditioned medium from hepatic differentiation of MSCs cultured in microfluidic device and 6-cm petri dish were collected at days 0 and 21 and stored at -20 ºC prior to the LDL and Urea uptake assays. LDL uptake of differentiated MSCs was assayed using the LDL Uptake Cell-Based Assay Kit (Cayman Chemical, Ann Arbor, Michigan, USA) according to manufacturer’s instructions. The urea concentrations were determined using the urea colorimetric assay kit (BioVision, Mountain View, CA, USA). Briefly, the urea standard curve was generated by diluting the urea standard (100mM) to generate 0, 1, 2, 3, 4, 5 nmol/well of urea. Next, 50μl of the conditioned medium from hepatic differentiation of MSCs cultured in microfluidic device and 6-cm culture dish were loaded in a 96-well plate, and mixed with the reaction mixture. The reaction mix per reaction contained 42 μl Assay Buffer, 2 μl Oxi Red Probe, 2 μl Enzyme, 2 μl Developer, and 2 μl Converter Enzyme. After incubating for 60 min at 37 ºC in the dark, the reaction was measured using the Infinite® M1000 PRO (Tecan, Männedorf, Austria) at an O.D. of 570 nm. Because the conditioned media collected for urea assay increased over time in the microfluidic system, we used pg/cell/hr as the unit for urea assay instead of pg/cell.

1.7. *Statistical analysis*

All experiments were conducted independently and represented as the mean ± SD (standard deviation). Statistical analyses were performed by using SPSS 17.0 software (IBM, San Rafael, CA, USA). Student t-test was used to determine the differences between the control group and the test group. For multiple groups, statistical differences were calculated using analysis of variance (ANOVA) of the turkey’s test. A P<0.05 was considered statistically significant.

1. **Supplementary table and figures**

**Table S1.** Sequence of qPCR primers for MSCs

| Gene | Sequence(5’ to 3’) |
| --- | --- |
| GAPDH  Forward  Reverse | GGGAAGCCCATCACCATCT  CGGCCTCACCCATTTG |
| AFP  Forward  Reverse | CACACCCGCTTCCCTCAT  CAAACTCATTTTCGTGCAATGC |
| TAT  Forward  Reverse | CGAGCCATTGTGGACAACAT  GGTCCCCAATTGACAGAGAT |
| CK18  Forward  Reverse | CCATGGACTCCGCAAGGT  CTTCCTTGAGTGCCTCGATTTCT |
| HNF4  Forward  Reverse | GAGGCTCCCCTGAGAATAGAGA  TGTTTGGTGTGAAGGTCATGATTAG |
| G6P  Forward  Reverse | GCCTCCGGAAGTATTGTCTCATC  CACCCCTAGCCCTTTTAGTAGCA |

**Figure S1.** The microfluidic device with larger culture chamber used for the study.


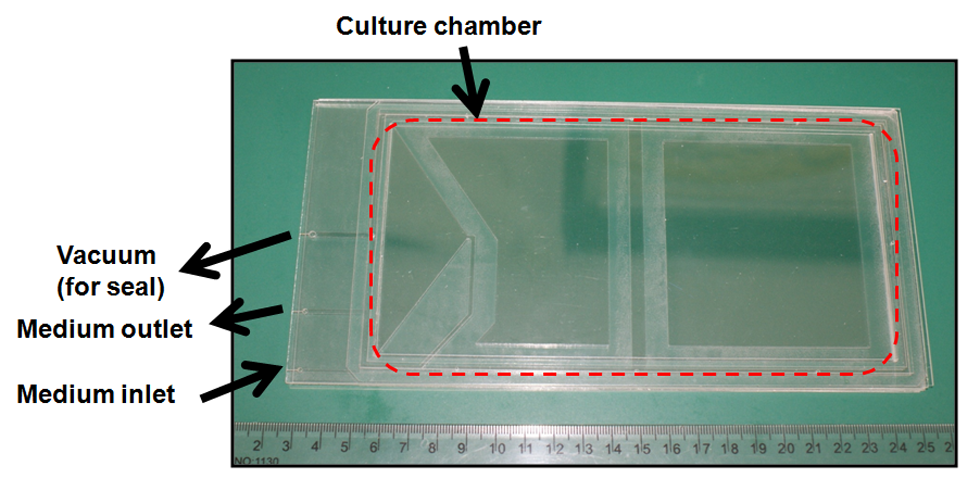


**Figure S2.** Processes of proliferation (A) and hepatic differentiation (B) of MSC in culture dish and microfludic device.


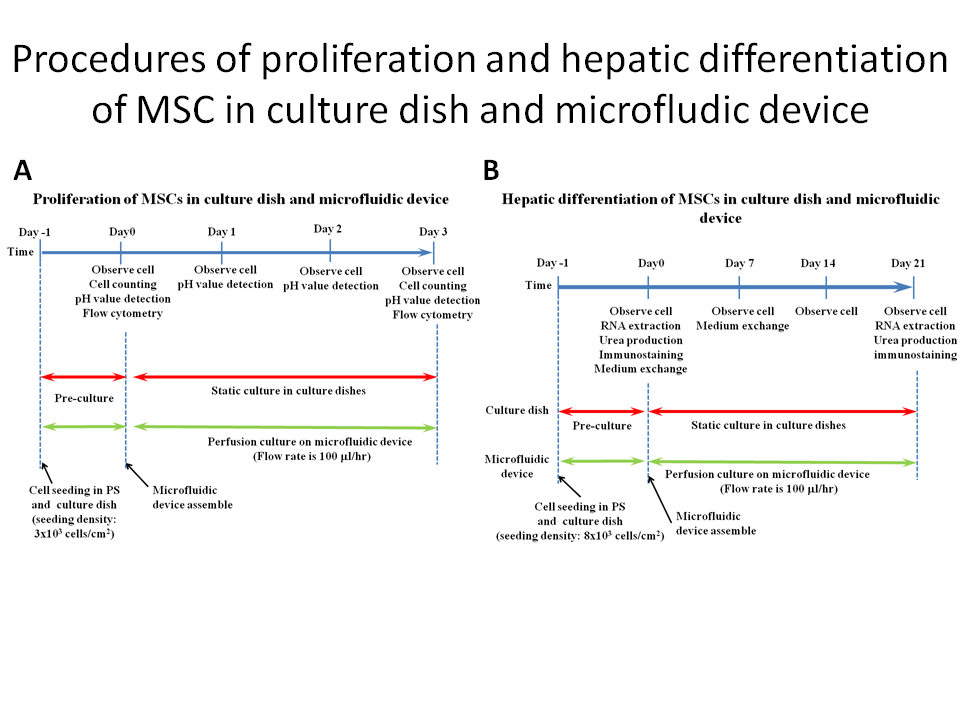


**Figure S3.** Growth curve of human MSCs cultured in the microfluidic device and culture dish from 0 to 9 days.


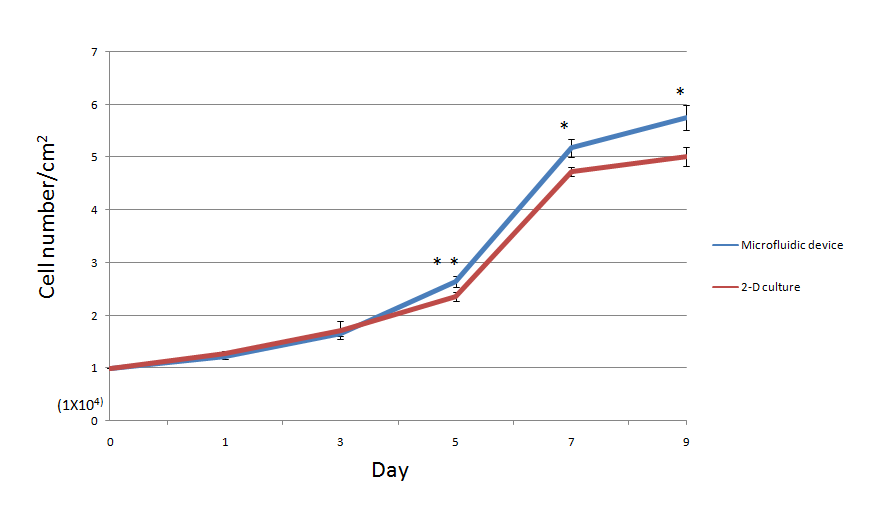


**Figure S4.** Comparative expression of surface markers in mouse MSCs cultured in static culture dish and microfluidic device at day 0and day 3


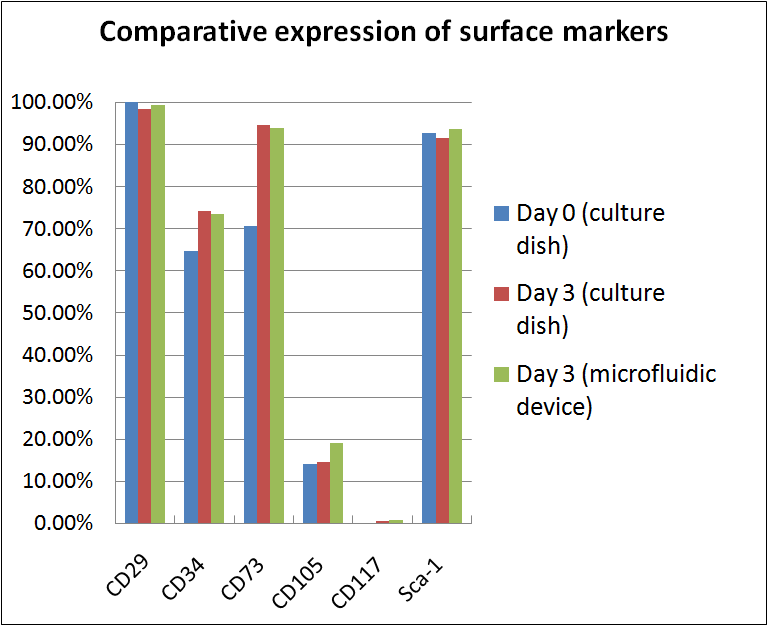


**Figure S5.** Simulation of culture medium diffusion in a circle cultural chamber. The flow field showed an uneven flow profile in a circle cultural chamber. The dimension and parameters of flow field were based from a previous study([Ju et al. 2008](#_ENREF_23)).

**
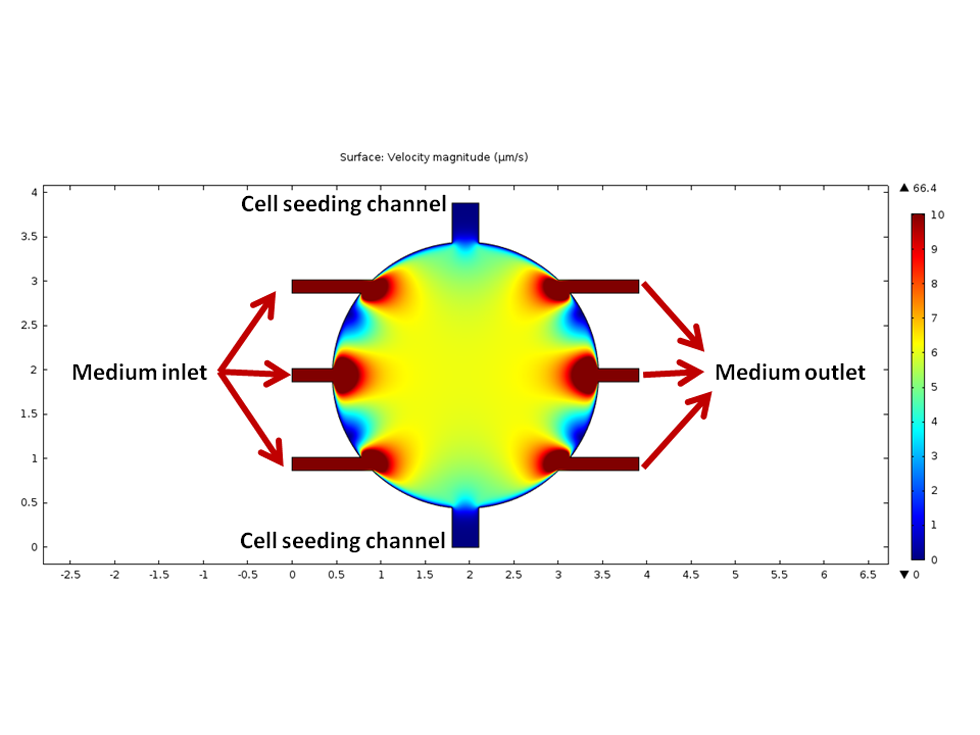
**
